# Geant4 Monte Carlo simulation study of the secondary radiation fields at the laser-driven ion source LION

**DOI:** 10.1038/s41598-021-03897-2

**Published:** 2021-12-24

**Authors:** M. Tisi, V. Mares, J. Schreiber, F. S. Englbrecht, W. Rühm

**Affiliations:** 1 Institute of Radiation Medicine, Helmholtz Zentrum München, Neuherberg, Germany; 2 Department of Medical Physics, Faculty of Physics, Ludwig-Maximilians-Universität, Garching bei München, Germany; 3 Max-Planck-Institute for Quantum Optics, Garching bei München, Germany

**Keywords:** Techniques and instrumentation, Plasma-based accelerators

## Abstract

At the Center for Advanced Laser Applications (CALA), Garching, Germany, the LION (Laser-driven ION Acceleration) experiment is being commissioned, aiming at the production of laser-driven bunches of protons and light ions with multi-MeV energies and repetition frequency up to 1 Hz. A Geant4 Monte Carlo-based study of the secondary neutron and photon fields expected during LION’s different commissioning phases is presented. Goal of this study is the characterization of the secondary radiation environment present inside and outside the LION cave. Three different primary proton spectra, taken from experimental results reported in the literature and representative of three different future stages of the LION’s commissioning path are used. Together with protons, also electrons are emitted through laser-target interaction and are also responsible for the production of secondary radiation. For the electron component of the three source terms, a simplified exponential model is used. Moreover, in order to reduce the simulation complexity, a two-components simplified geometrical model of proton and electron sources is proposed. It has been found that the radiation environment inside the experimental cave is either dominated by photons or neutrons depending on the position in the room and the source term used. The higher the intensity of the source, the higher the neutron contribution to the total dose for all scored positions. Maximum neutron and photon ambient dose equivalent values normalized to 10^9^ simulated incident primaries were calculated at the exit of the vacuum chamber, where values of about 85 nSv (10^9^ primaries)^−1^ and 1.0 μSv (10^9^ primaries)^−1^ were found.

## Introduction

Thanks to the recent improvements in laser peak power, energy density, laser temporal contrast and to the large investigation of suitable target materials, in the last two decades, several groups achieved the acceleration of protons and light ions up to an energy of several tens of MeV^1^. Although still in an early phase of its development, the acceleration of charged particles via laser-target interaction is becoming, in the present days, a highly promising candidate for future acceleration techniques^2^. Due to the micrometer scales at which the acceleration process takes place, laser-driven ion sources are ideally suited for novel investigations in research and have high potential for pushing the frontiers for future generations of particle accelerators for a broad range of applications that benefit from short ion bunch duration and high peak current^3^.

At the Center for Advanced Laser Applications (CALA) located in Garching close to Munich (Germany), the laser-driven ion acceleration experiment LION is currently being commissioned^4^.

As laser pulse source, LION employs ATLAS3000, a Ti:Sapphire-based laser, whose main properties are summarized in Table 1, and as targets, 0.01–1 μm thick metal or carbon-based samples mounted on a rotating sample holder^5^. The ultimate goal pursued at LION is the exploitation of the Target Normal Sheath Acceleration (TNSA) and Radiation Pressure Acceleration (RPA) regimes, in order to realize a laser-driven ion source with the capability to deliver collimated bunches of several tens of MeV ions (protons and carbon ions) at 1 Hz repetition frequency to serve as a facility for radiation therapy research^6^.

**Table 1:** Main ATLAS3000 laser parameters available for the LION experiment, considering full operation and current status.

	Laser pulse energy *E* (J)	Duration *t* (fs)	Repetition frequency *f*
Full operation	60	20	1 Hz
Current status	< 10	30	Shot on demand

As reported in Table 1, at the current status of the facility’s commissioning (2020), ATLAS3000 is delivering to LION laser pulses with energy up to 10 J. This results in ion cutoff energies (i.e., the energy of the most energetic ions, detected above the background noise) and charge per bunch that are lower to those expected at full operation.

Computational investigations of the secondary particle production expected at LION during its commissioning, and afterwards in full operation, are of primary interest for a variety of reasons. Radiation protection is typically the primary purpose. In order to protect the accelerator operators and the general public from being exposed to an unwanted dose of secondary particles, a realistic dose assessment based on simulations (followed by experimental verification) needs to be done. To date, different studies aiming at assessing the unwanted secondary neutron radiation produced by laser-driven ion sources appeared in the literature. Fan et al.^7^ addressed the problem of unwanted secondary radiation through designing a multi-layer compact radiation shielding for a laser-driven proton therapy facility using FLUKA Monte Carlo code. Secondary neutron doses per primary protons are given in the proximity of the vacuum chamber, assuming a primary proton spectrum with 300 MeV cutoff energy. In 2010 Sakaki et al. presented a combined experimental and PHITS Monte Carlo study of the secondary doses produced at KPSI’s (Kansai Photon Science Institute) laser-driven proton source, showing a few μSv of total dose per bunch were found in the proximity of the facility’s vacuum chamber^8^. Radiation protection oriented FLUKA simulations for the several facilities hosted at CALA (among which LION appears as well) have been recently reported by Englbrecht et al.^9^. This study demonstrates that radiation protection limits in all areas of interest around the facilities are met, even for worst case scenarios (in terms of the energy spectrum of primary particles, charge per bunch and repetition frequency). Considering LION only, it has to be pointed out that elements and structures hosted inside the LION vacuum chamber, where a significant fraction of secondary particles gets produced due to the large divergence angles of laser-driven emitted particles, are not included in the work of Englbrecht et al. From a radiation safety point of view, this has little influence given that access to the target area is not permitted during operation. In contrast, our inclusion of more details mainly serves the purpose of evaluating the secondary radiation fields, which can be relevant for studies with particle bunches inside the LION cave. In this sense, a clear characterization of the secondary radiation produced in the vicinity of the particle acceleration is of interest when planning future experimental applications (e.g., radiobiology experiments), where the contribution to the dose due to secondary neutrons and photons produced by the shaping apparatus might be non-negligible if compared to the dose delivered by primary protons.

The production of secondary radiation is directly connected to the presence and the interaction of primary radiation (i.e., laser-accelerated particles) with surrounding materials and structures. A detailed information on the nature of the secondary radiation might lead to a deeper knowledge of the specific features of the primary radiation that was responsible for its production (e.g., number of primary particles per bunch and angular distribution), specially when only a small portion of the produced primary particles can be directly detected and analyzed.

Lastly, simulations of the secondary pulsed radiation fields, expected during the different stages of LION commissioning, will drive the decision on which neutron and photon detection techniques it is best to apply when experimental characterizations of the secondary radiation will be performed. This is even more relevant considering the pulsed nature of the LION source and the serious issues encountered by commercial radiation protection online devices when exposed to pulsed neutron and photons sources^10,11^.

## CALA and the LION experiment

Laser-driven acceleration is a quasi-neutral acceleration process that transfers a fraction of the energy carried by laser photons to kinetic energy of a variety of particles, first of all electrons and light ions (protons mainly). These propagate in forward direction, from the laser-target interaction site, with a relatively wide diverging angle of the order of a few hundreds of mrad^12,13^.

During the current commissioning phase, the production and transport of protons dominates that of other ions. In the following, we will therefore refer only to the laser-driven production of protons and electrons, neglecting the small, yet present, contribution of other ions to the ensemble of laser-driven produced particles.

The acceleration process takes place within a 2.5 cm thick aluminum vacuum chamber, a modular structure 3.92 m long, 1.21 m high and 0.98 m wide. The vacuum chamber itself is located inside the LION cave, an experimental cave 18 m long, 3 m wide and 4.25 m high, separated from the ground by a 75 cm thick concrete platform. One meter above the platform lies a double floor, below which, part of technical infrastructures is hosted. In addition, the cave is covered by a 45 cm thick concrete ceiling.

**Figure 1:**
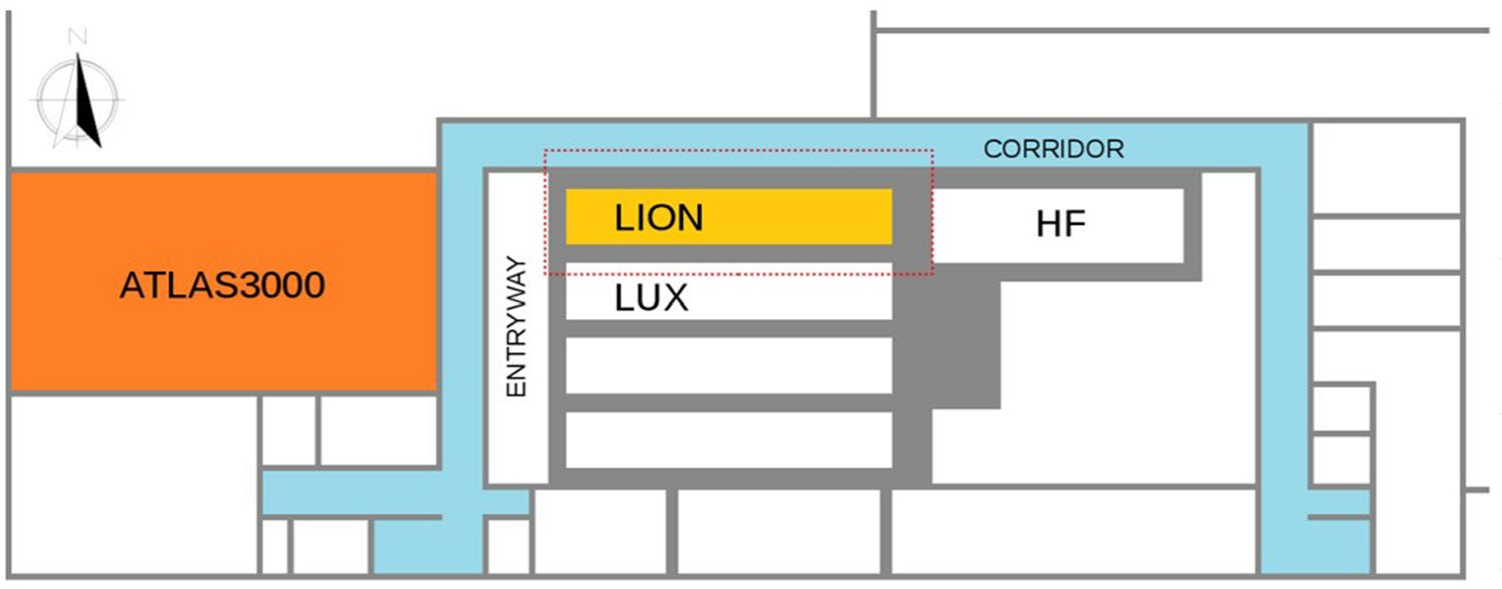
Top-view drawing of the CALA building. In orange the room hosting ATLAS3000 laser, in yellow the LION cave and in light-blue the corridor surrounding the facilities’ caves. Names of rooms adjacent to the LION cave are reported: *HF* high field, *LUX* laser-driven Undulator X-ray source. The area enclosed within the red dashed line is the one considered by the Geant4 simulations shown in this work.

**Figure 2:**
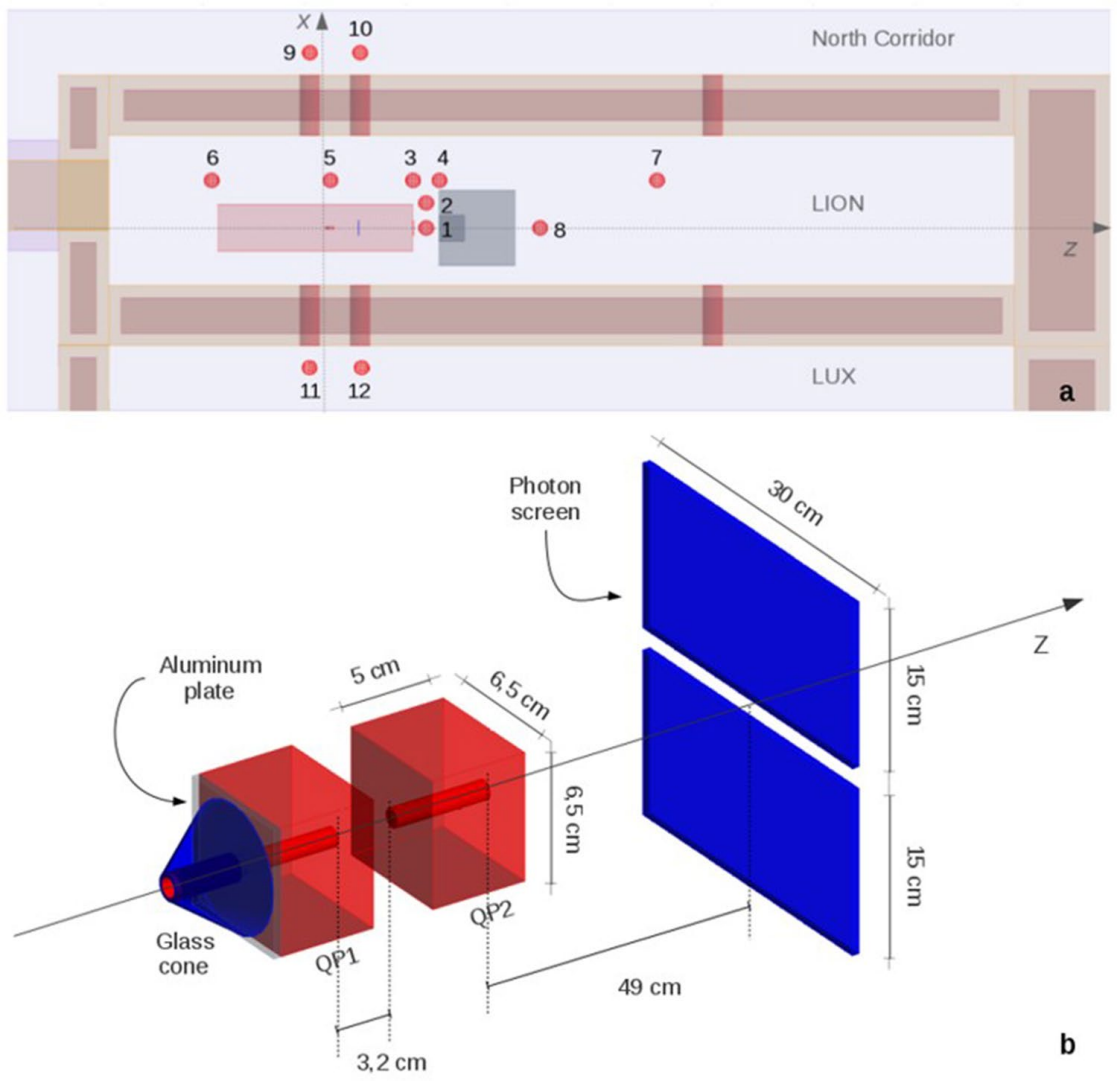
LION geometry as implemented in Geant4. (**a**) Top view of the facility and scorers (red) with ID number. (**b**) Close-up on the two quadrupole magnets (QPs), with protection layer and glass hollow cone mounted on the first QP and photon screen. Figures are not drawn to scale.

As shown in Fig. 1, LION is surrounded to the south by the LUX cave (Laser-driven Undulator X-ray Source), to the east by the HF cave (High Field), to the west by the facilities’ entryway (closed during operation), and to the north by a corridor whose access is granted to operators during machine operation. To separate LION from these other areas and to shield them from possible secondary radiation, radiological shielding walls with a thickness ranging from 1 to 1.2 m are in place (exception is the east wall whose thickness reaches up to 2 m). Shielding walls are weakened by the presence of six cylindrical openings 40 cm in diameter (Fig. 2a), which are needed to transport laser light from ATLAS3000 to the LION experiment and then further to the other experimental installations. These openings are placed between the concrete platform and the double floor, three of which connecting the LION cave with the LUX cave (through LION’s southern wall) and three connecting the LION cave with the corridor through LION’s northern wall.

As a result of radiation protection oriented FLUKA simulations, performed in an early phase of the experiment commissioning, a composite water-concrete beam dump is placed in front of the back exit of the vacuum chamber (about 50 cm from the chamber), in order to absorb that fraction of produced protons that is transported outside of the vacuum chamber (Fig. 2a). This beam dump also acts as radiation shielding for the shower of secondary particles (photons and neutrons mainly) that are produced by the interaction of the particles emerging from laser-target interaction with the diagnostic and steering components hosted inside the vacuum chamber^9^.

As mentioned, protons produced via laser-target interaction are usually emitted with a large divergence angle. This is usually, from an application point of view, a quite inconvenient feature, given that, in the majority of applications, the delivery of particles needs to be precisely focused onto a specific target volume. Therefore, following the laser-target interaction site, a series of two NdFeB quadrupoles (later referred to as QPs) mounted on motorized supports, is employed to focus the produced protons. Given the large divergence angle at which protons are intrinsically emitted in this facility, around 180 mrad half angle^14^, most of protons interacts with the front face of the first QP itself (which is, also for this reason, shielded by a 4 mm thick aluminum protection plate) rather than passing through it and getting focused by the applied magnetic fields. The fraction of protons that gets focused by the QPs travels straight and leaves the vacuum chamber through a thin exit window after which proton diagnostic devices are placed (such as radiochromic stacks, transmission chambers or scintillation foils). These protons are eventually stopped by the aforementioned beam dump.

## Materials and methods

For the simulation of the production of secondary radiation at LION, the Geant4 10.1.2 Monte Carlo simulation toolkit has been used^15,16^. Given that the interaction of multi-MeV protons and electrons (also referred to as *primary particles*) with surrounding materials leading to the production of secondary neutrons and photons (*secondary particles* in the following) is the main focus of this work, the Bertini Intranuclear Cascade model (QGSP_BERT_HP) has been used. This model takes into account hadron physics, electromagnetic showers and synchrotron radiation, and it is recommended by the Geant4 developers when dealing with medical and industrial neutron applications and radiation shielding^17^. The HP extension (i.e., NeutronHP, Neutron High Precision) accurately describes the transport of neutrons from 20 MeV down to thermal energies.

An unambiguous reference frame has been defined to locate simulation elements within the geometry. The coordinates’ origin lies at the proton production site, which is therefore at (0,0,0), and the proton bunches propagate by definition in the direction of positive z.

### Geometry

During the present commissioning phase, in the LION vacuum chamber several different components are required in order to produce and deliver laser-driven protons with desired features. For our purpose, only those elements directly interacting with the primary particles are of interest and, hence, only these were included in the simulation environment. This approach allowed to take into account only those elements that play a key role in the production of secondary particles, neglecting all structures that might introduce only small differences in the secondary particle production. This made possible reducing both modeling and computation times significantly. As shown in Fig. 2b, these elements are the two QPs (red) and a glass-made photon screen used for laser light diagnostics (blue).

QPs are modeled as two parallelepipeds 6.5 × 6.5 × 5 cm^3^ with a circular hole 1 cm in diameter oriented along the z axis. The first QP lies at 4.7 cm from the proton source and the second one at 7.9. As in reality, they are made of a NdFeB alloy (density = 8 g/cm^3^).

The photon screen consists of two 1 cm thick glass slabs, separated by a few centimeters gap (Fig. 2b) and positioned at about z = 67 cm.

To avoid radiation damages leading to a possible demagnetization of the QPs, the front face of the first QP is shielded by a 4 mm thick aluminum protection plate (density = 2.7 g/cm^3^) depicted as a gray slab in Fig. 2b. In addition, a hollow cone (made of a particular borosilicate glass of 2.23 g/cm^3^) is mounted on the protection plate. Its main purpose is to avoid possible laser light back reflections from the aluminum protection plate surface. This detail has been included because the most divergent protons can interact with this cone before reaching the aluminum protection plate.

As a general remark, all simulations include the presence of the LION cave walls (with laser openings included) and the beam dump with realistic dimensions and material composition in order to properly quantify any back-scattered radiation. The structure of the vacuum chamber has been simplified from reality, by omitting the presence of all technical openings (closed by aluminum flanges) and its stainless-steel structure. For simplicity, the chamber has been modeled as parallelepiped with 2.5 cm thick aluminum walls. In our view, these approximations do not strongly affect the production of secondary particles, given that technical openings, as said, are also in reality closed by aluminum flanges with similar thickness, and that the stainless-steel structure, on which the 2.5 cm thick aluminum plates are mounted, has negligible dimensions and is not directly in the way of the produced particles.

### Scored quantities

Eight spherical neutron and photon track length scorers (30 cm diameter, numbered from 1 to 8) have been placed throughout the LION experimental cave at y = 0 (Fig. 2a). Their positions have been chosen in order to derive a neutron and photon dose mapping of the LION cave. Scorers 1 and 2 are at the back exit of the vacuum chamber where a hot-spot is expected. Scorers from 3 to 7 are placed at about 1 m from the z-axis and scorer 8 is behind the beam dump on the z-axis. Special attention is given to scorer 5 that is placed at z = 0, same z position as the source term.

For radiation protection purposes, four scorers (9–12), in addition to the ones mentioned above, are placed outside the LION cave near the exit of the four laser openings located close to z = 0, where higher dose rates are expected due to their vicinity to the source of secondary radiation. These are positioned a few centimeters above the double floor at locations where people might stand during beam operation. All scorers are depicted in Fig. 2a as red spheres with their respective ID number.

Neutron and photon scorers include 132 energy bins, 10 bins per decade, logarithmically equispaced from 8.91 × 10^–10^ to 1.12 × 10^4^ MeV. The output of each scorer is the secondary particle fluence per primary particle expressed in cm^−2^. Neutron and photon doses per primary can be derived by folding the neutron and photon fluence spectrum with the fluence to ambient dose equivalent (H*(10)) conversion coefficients for neutrons and photons^18^. In the following, it has been decided to normalize both fluence and dose values to 10^9^ protons or electrons.

### Primary particle energy spectrum

#### Protons

In order to best characterize the secondary fields that are to be expected at LION and derive realistic results, the approach followed in this work is to use, as primary source terms, three different experimentally found proton spectra taken from literature that are representative of three specific commissioning phases of the LION experiment:

- Zeil2010^19^: Proton spectrum currently available (commissioning phase 1, 300 TW).
- Ma2019^20^: Proton spectrum reachable within a couple years (approachable with 1 PW).
- Wagner2016^21^: Proton spectrum reachable within three to five years (approachable with 3 PW and loose focus, i.e., not optimized for highest maximum energy).

Proton spectra numerical values have been taken from^22^ where they are given as number of protons per unit energy and unit solid angle. Each value represents the number of protons of energy *E* in a bin of width 1%*E* centered around energy *E*, per millisteradian (msr). To these experimental data points an exponential regression has been applied. This latter has been then sampled in order to create a spectrum that covers energies from 1 MeV to the cut-off energy, $E_{ion,cutoff}$, of each dataset and where each bin $i$ is centered at energy $E_{i}$ and has a width of $1\% E_{i}$, for a total of 250 bins for *Zeil2010*, 404 bins for *Ma2019* and 445 bins for *Wagner2016*.

**Figure 3:**
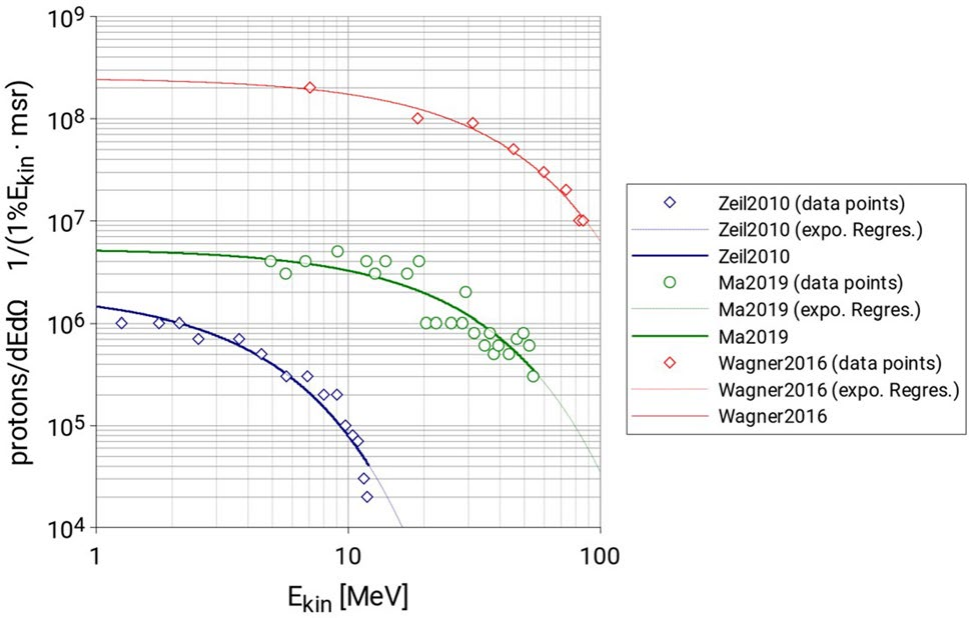
Primary proton spectrum data as reported by Schreiber in^22^ with exponential regression applied (dashed lines). Proton spectra found applying the exponential regression to the proton spectrum data (solid curves).

**Figure 4:**
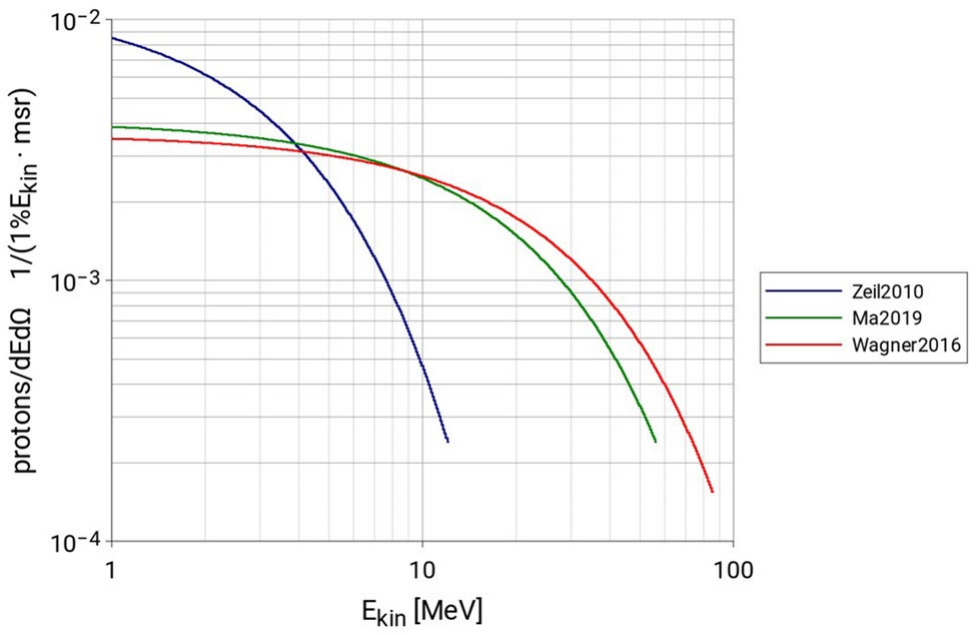
Proton spectra (normalized to integral = 1) used as input parameter for the LION-Geant4 simulations.

Figure 3 shows the experimental proton spectra produced at laser-driven proton sources as reported in^22^, together with results of exponential regressions of these spectra. Figure 4 shows the relative primary proton spectra (the value of each bin *i* is the probability of having a proton with energy *E*_*i*_) which are used as input files for the Geant4 Monte Carlo simulations.

#### Electrons

It is worth mentioning here that we are not aware of any measured electron energy distributions in parallel to the proton energy distributions. In future, such measurements (as proposed, for example in Lindner et al.^23^) could provide valuable input to more accurate simulations. Therefore, within this work, a simple model of an exponentially shaped electron source is used. The number of electrons $N_{{e^{ - } }}$ of energy $E$ follows Eq. (1):

1 $$ N_{{e^{ - } }} (E) = \frac{{N_{{e^{ - } ,0}} }}{{E_{{e^{ - } ,0}} }}e^{{ - \frac{E}{{E_{{e^{ - } ,0}} }}}} $$

where $E_{{e^{ - } ,0}}$ (also referred to as electron temperature) is derived from Eq. (2)^24^:

2 $$ E_{ion,cutoff} = \left( {4.6E_{{e^{ - } ,0 }} \pm 287} \right) \;keV $$

$$E_{ion,cutoff} $$ is the measured cut off energy of the corresponding proton spectrum. Table 2 summarizes the ion cut-off energies for each spectrum and the respective electron temperatures.

**Table 2:** Summary of proton cutoff energies and electron temperatures of the particle spectra used.

Proton spectrum	$E_{ion,cutoff}$ (MeV)	$E_{{e^{ - } ,0 }}$ (MeV)
Zeil2010	11.92	2.59
Ma2019	56.05	11.96
Wagner2016	85.93	18.68

To reproduce the quasi-neutrality of the plasma condition, the total number of electrons considered in this work equals the number of simulated protons.

3 $$ N_{{e^{ - } ,tot}} = N_{ion,tot} $$

**Figure 5:**
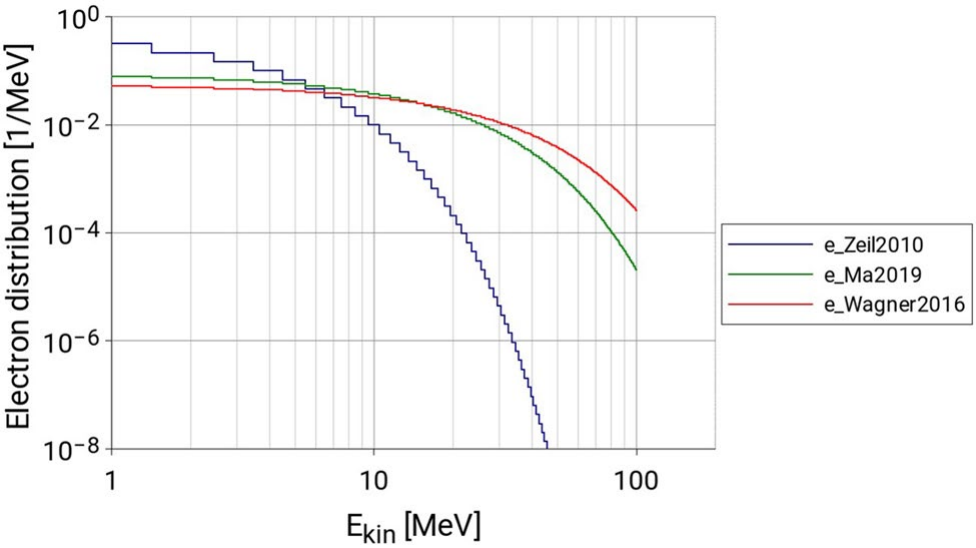
Relative electron spectra used as input parameter for the LION-Geant4 simulations.

As shown in Fig. 5, electron distributions have been implemented as probability step curves with 1 MeV wide bins, from 1 to 100 MeV.

### Angular distribution

Particles produced via laser-target interaction are usually emitted with a large divergence angle whose value ranges from a minimum of zero to a maximum of a few hundred mrad^25^. As mentioned, a few centimeters away from the laser-target interaction site, two QPs focus the diverging protons in order to collimate the proton bunch. Since the QPs’ acceptance angle is lower than the proton initial divergence, it is assumed that about 90% of protons hits the front face of the first QP and only the 10% pass through them and are transported. Although protons with high energy tend to be more forward-peaked compared to protons with low energy^26^, as a first order approximation we neglect this feature and assume that the spectrum of particles does not vary over the emission angle.

**Figure 6:**
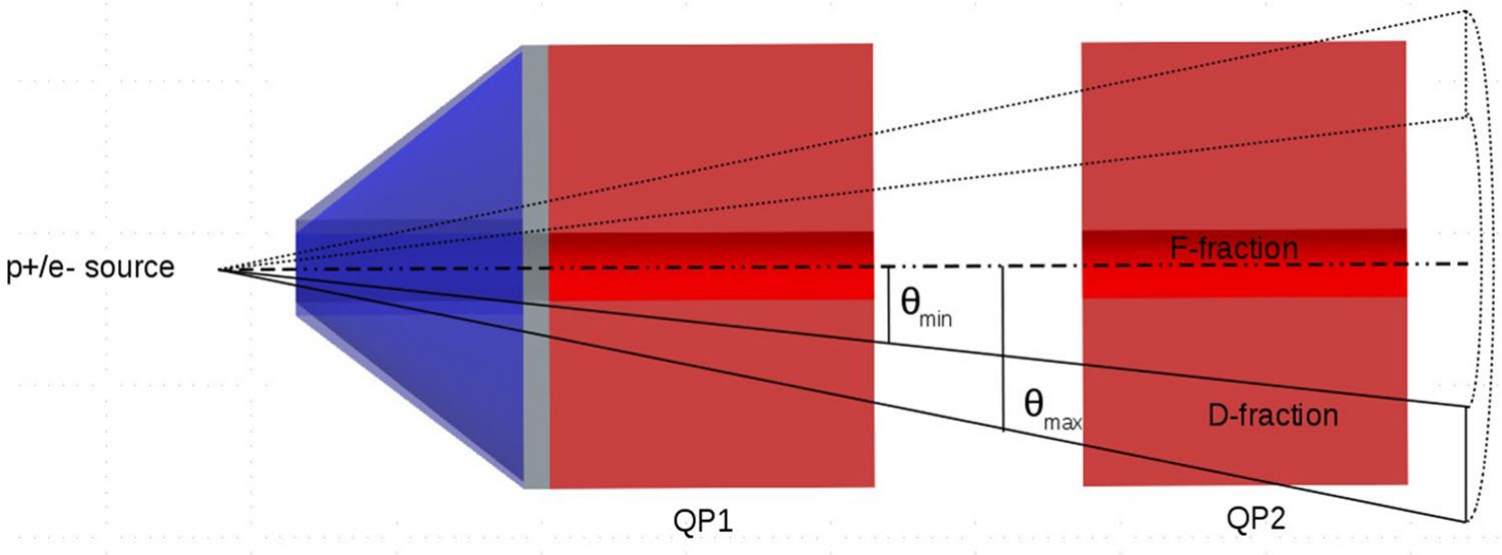
Proton and electron source geometrical model. Divergent fraction (D-fraction, within solid lines) and focused fraction (F-fraction, dash-dotted line).

To take into account the focusing action exerted on protons by the QPs, the proton source term has been modeled in a way that the fraction of protons that transmits through the QPs continues as a focused beam (*F-fraction*) through the QPs, while the fraction of protons that does not get focused diverges with a diverging angle between $\theta_{max}$ and $\theta_{min}$. Where, as shown in Fig. 6, $\theta_{max}$ equals 180 mrad and $\theta_{min}$ is the smallest angle at which protons still interact with the front face of the first QP. We denote this second component as divergent fraction (*D-fraction*) for simplicity. Monte Carlo simulations were run for the two components separately and their results were afterwards linearly superimposed with the given proportions. Thanks to this simplified approach, no magnetic fields need to be included into the Geant4 simulation and this benefits lower simulation times.

Along with protons, also a fraction of electrons transmits through the QPs, for this reason a similar 2-components source term is used to model electrons. It is assumed, that the 90% of electrons belongs to D-fraction and 10% belongs to F-fraction, in analogy with the proton source term. Given the lack of knowledge on the actual electron divergence, it is moreover supposed that the maximum electron divergence equals the maximum proton divergence of 180 mrad. It needs to be mentioned that a small fraction of protons is emitted in backward direction. As shown by Ter-Avetisyan et al*.*^27^ this component shows a lower cutoff energy (about a factor 2) and a significantly lower charge per bunch compared to forward-emitted protons. For this reason, we decided to neglect its contribution to the total number of emitted particles in the present work.

### Secondary neutron sources

Proton-induced neutron production reactions are threshold reactions, whose threshold values depend on the nucleus involved and on the specific reaction considered, such as (p,n), (p,2n), (p,n + ^3^He) or (p,n + α). Other nuclear reactions might happen, leading to the production of more than two neutrons per event, but these have usually higher thresholds making them quite unlikely to happen given the primary proton spectra employed.

Based on the geometrical definition of the source and the components that surround it, in the current commissioning phase, three main locations for the production of secondary neutron radiation are expected.

The first source of neutrons are the glass cone and the aluminum protection plate that shields the first QP, where 90% of protons deposit their energy. Here protons with the lowest energy are stopped by the 2 mm thick glass layer of the hollow cone, while the most energetic ones can reach the aluminum plate.

Borosilicate glasses are usually composed of about 35% of silicon in mass fraction, 55% of oxygen and around of 5% boron. The rest are minor components such as, aluminum, sodium and nitrogen.

Regarding silicon (considering ^28^Si only, that accounts for 92.2% of natural silicon), the first reaction channel that leads to a neutron as output is ^28^Si(p,^3^He + n)^26^Al, whose reaction threshold is around 9 MeV. For oxygen (^16^O almost 100% abundance in natural oxygen), at around 6 MeV, a few reaction channels open up leading to the production of neutrons such as: ^16^O(p,^3^He + n)^13^N, ^16^O(p,n + p)^15^O and ^16^O(p,n + d)^14^O. Even though boron accounts only for a small fraction, it’s worth mentioning that ^11^B (around 90% abundance in natural boron) shows a 3 MeV cross section threshold for the ^11^B(p,n)^11^C reaction.

The protection plate is made of aluminum (^27^Al, 100% abundance in natural aluminum) which has a (p,n) cross section threshold enabling the production of neutrons at about 5.8 MeV.

Protons with energy lower than a few MeV will lose their energy via scattering and ionization without producing neutrons.

When protons (belonging to F-fraction) leave the vacuum chamber, they enter into the 50 cm wide air gap (which represents the second source of neutrons) present between vacuum chamber and beam dump. According to ICRU Report 90^28^, protons with energy lower that 6 MeV get stopped before reaching the beam dump (6 MeV proton range in air ≈ 48 cm). Here, reactions with ^16^O and ^14^N are the most probable. The (p,n + ^3^He) reaction channel on ^14^N opens at about 2.5 MeV and has its maximum cross section at around 10 MeV, while the (p,n) reaction channel opens at 6.3 MeV.

The third source of secondary neutron radiation is the beam dump, where the F-fraction of protons with initial energy above 6 MeV gets dumped after leaving the vacuum chamber and crossing the air gap. Protons entering the beam dump first encounter the 50 × 50 × 50 cm^3^ water-made insert of the composite beam dump. Given that the most energetic protons considered in this work reach 85 MeV and that 100 MeV proton range in water is around 7.8 cm^28^, no proton will directly reach the concrete part of the beam dump. Therefore, regarding the beam dump, the production of neutrons depends almost entirely on the oxygen of water. Main reaction channels, threshold energies and maximum cross sections are summarized in Table 3.

**Table 3:** Summary of the main proton reaction channels leading to neutron production with threshold energies and maximum cross sections, ordered according to the atomic number of the target element.

Reaction	Threshold energy (MeV)	Max. cross section
^11^B(p,n)^11^C	3	175 mb (at 12 MeV)
^14^N(p, n)^14^O	6.3	2 mb (at 20 MeV)
^14^N(p, ^3^He + n)^11^C	2.5	130 mb (at 10 MeV)
^16^O(p,^3^He + n)^13^N	6	50 mb (at 10 MeV)
^16^O(p,n + p)^15^O	6	100 mb (at 30 MeV)
^16^O(p,n + d)^14^O	6	1 mb (at 50 MeV)
^27^Al(p,n)^27^Si	5.9	85 mb (at 14 MeV)
^28^Si(p, ^3^He + n)^25^Al	9	40 mb (at 16 MeV)

As a general comment, given the rather low proton energies considered in this work (< 85 MeV), spallation reactions will play a minor role^29^.

As mentioned above, the electron source term is modeled in the same way as the proton source term. We therefore expect the production of secondary particles to happen at the same locations.

Electrons lose their energy mainly through radiation-less collisions and *bremsstrahlung* (radiative losses). The so-called critical energy, *E*_*c*_, defines the threshold where bremsstrahlung losses equal radiation-less losses. This is often derived from the well-known relation $E_{c} \;[MeV] = 800{/}(Z + 1.2)$^30^. Electrons with energy $E \gg E_{c}$ traveling through media are likely to initiate an *electromagnetic shower* in which electrons, positrons and photons continuously transform and loose energy while interacting with the medium. This electromagnetic shower stops when the electron energy falls below *E*_*c*_ and radiation-less collision start dominating the energy loss process again.

Bremsstrahlung photons produced in such a way and with energy above a certain threshold (whose typical value is in the range of 6–8 MeV) can undergo photo-nuclear reactions (γ,n). For photon energies below 30 MeV, the dominant photo-neutron production mechanism will be through photo-nuclear giant resonance^31^, a nuclear collective excitation mode that leads to the emission of quasi-isotropic neutrons with a nuclear evaporation-like spectrum. For higher energies ($30\;MeV < E_{\gamma } < 300\;MeV$) pseudo-deuteron production is most likely to occur, where the gamma photon interacts with a neutron-proton pair inside the nucleus rather than with the nucleus as a whole (detailed information can be found in^32^). Also direct electronuclear reactions, that lead to the production of neutrons can happen, but their probability is about two orders of magnitude smaller than the corresponding probability for photons^33^.

It is furthermore noticeable, given the geometrical model used to simulate the source of primary particles (where all particles either interact with the QPs or pass through them), that no primaries directly interact with the vacuum chamber. In reality a minor fraction of laser-driven particles could also be emitted with even larger angles. Such particles would then interact directly with the vacuum chamber walls and give rise to an additional source of secondary radiation.

### Code benchmark

A comparison with FLUKA simulations has been performed in order to benchmark the Geant4 environment developed for this study. By removing the structures located inside the vacuum chamber (QPs and photon screen) and omitting the aluminum flange placed on the downstream face of the vacuum chamber, the Geant4 geometry of this study and the FLUKA geometry one described by Englbrecht et al. in^9^ are identical. As source term, primary protons with a box-like energy spectrum (where the probability to have a proton in an energy bin is kept constant over the whole energy range) ranging from 10 to 75 MeV and with 180 mrad total divergence were used. In both FLUKA and Geant4, neutron fluence spectra have been acquired in scorers from 1 to 8 (as described in the “Materials and methods” section). As shown in Table 4, an average relative difference of the total neutron fluence of 21% was found, allowing us to conclude that the two simulation environments show a reasonable agreement.

**Table 4:** Intercomparison between Geant4 and FLUKA MC codes using a primary source term composed by protons with energies ranging from 10 to 75 MeV. Neutron fluence results are given in cm^−2^proton^−1^ for each scored position. The last column shows the ratio between the results of the two codes.

Scorer ID	Neutron Fluence [cm^−2^ protons^−1^]		Relative difference (%)
	Geant4	FLUKA	
Scorer 1	7.18 × 10^−7^	6.05 × 10^−7^	19
Scorer 2	1.79 × 10^−7^	1.42 × 10^−7^	26
Scorer 3	4.09 × 10^−8^	3.11 × 10^−8^	32
Scorer 4	1.93 × 10^−8^	1.75 × 10^−8^	11
Scorer 5	1.79 × 10^−8^	1.34 × 10^−8^	34
Scorer 6	8.93 × 10^−9^	5.98 × 10^−9^	49
Scorer 7	5.38 × 10^−9^	4.67 × 10^−9^	15
Scorer 8	2.33 × 10^−9^	2.04 × 10^−9^	14
Average			21

## Results

The results section is divided into two main parts: the first one describes the simulated neutron fluence spectra per 10^9^ primary protons and electrons, for all scored positions inside the LION cave, whilst the second one shows neutron and photon doses per 10^9^ primaries for all scorers.

### Proton- and electron-induced neutron fluence

Generic considerations on the neutron spectra expected at LION can be outlined looking at neutron spectra measured in the proximity of proton and electron accelerators.

In general, neutron fluence spectra detectable in the proximity of proton accelerator facilities show some well-recognizable typical features: a Maxwell–Boltzmann peak centered at thermal energies, a *plateau* in the epi-thermal neutron region (E_n_ < 100 keV) and a peak around 1–2 MeV, generated by the nuclear evaporation process of the nuclei involved in the proton-induced nuclear reactions. In case of high-energy primary protons (E > 20 MeV), also a high-energy neutron peak with energy comparable to the primary maximum energy can be found^34,35^.

Neutron spectra around electron accelerators are dominated by a pronounced peak at around 1 MeV due to the evaporation of neutrons as a consequence of the giant dipole resonance interaction^36^. As it was described for proton accelerator facilities, similar Maxwell–Boltzmann peak and epi-thermal neutron *plateau* regions are expected*.*

#### Zeil2010 p^+^ and e^−^ sources

**Figure 7:**
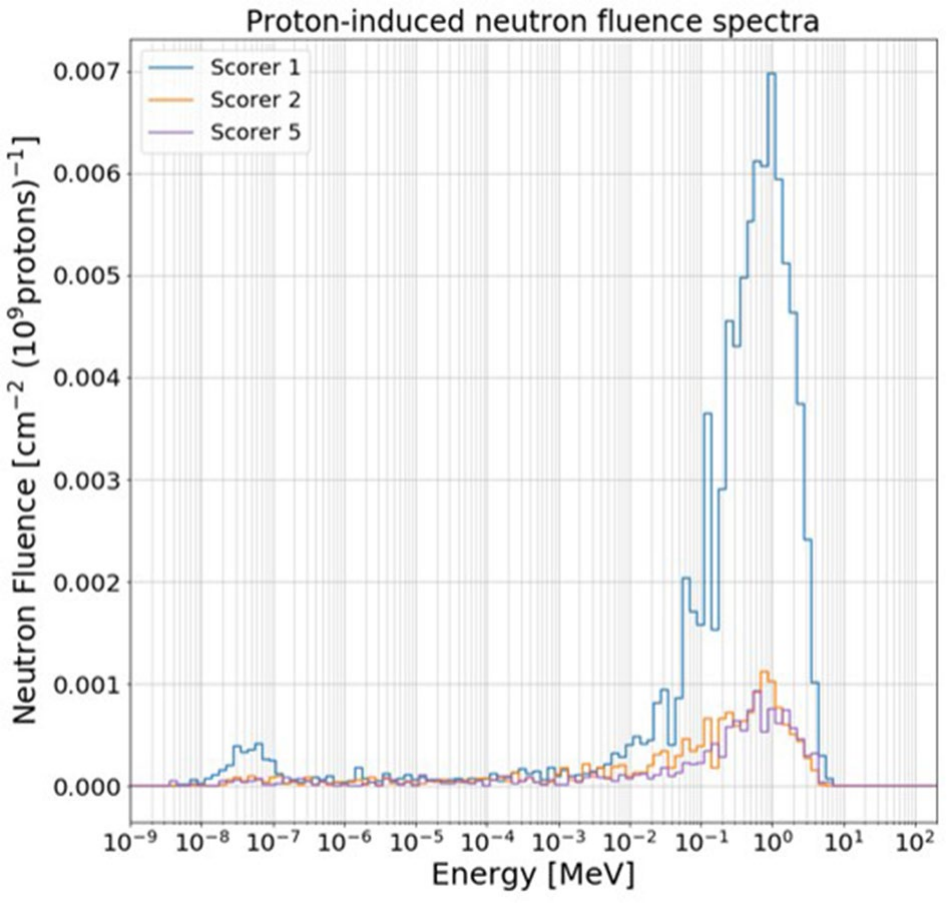
Proton-induced neutron fluence spectra for scorers 1, 2 and 5 (see Fig. 2) using Zeil2010 input parameters. Values are normalized to 10^9^ primary protons.

Zeil2010 primary proton and electron source terms are the ones that have the lowest cut off energy and steepest energy-dependent particle spectrum among the primary particle sources considered in this study (see Figs. 3 and 4). The total neutron fluence per 10^9^ primaries in all scorers inside the LION cave resulted to remain below 10^–1^ and 10^–2^ cm^−2^ (10^9^ primaries)^−1^ for primary protons and electrons respectively. In Fig. 7 it has been decided to plot neutron spectra only for those scorers that showed a total neutron fluence higher that 10^–2^ cm^−2^ (10^9^ primaries)^−1^ (namely, scorers 1, 2 and 5 for the proton source case) because of statistical reasons. Well recognizable in Fig. 7 is the neutron evaporation peak for all three scored positions. Total neutron fluences can be easily calculated by summing the values in each bin.

#### Ma2019 p^+^ and e^−^ sources

**Figure 8:**
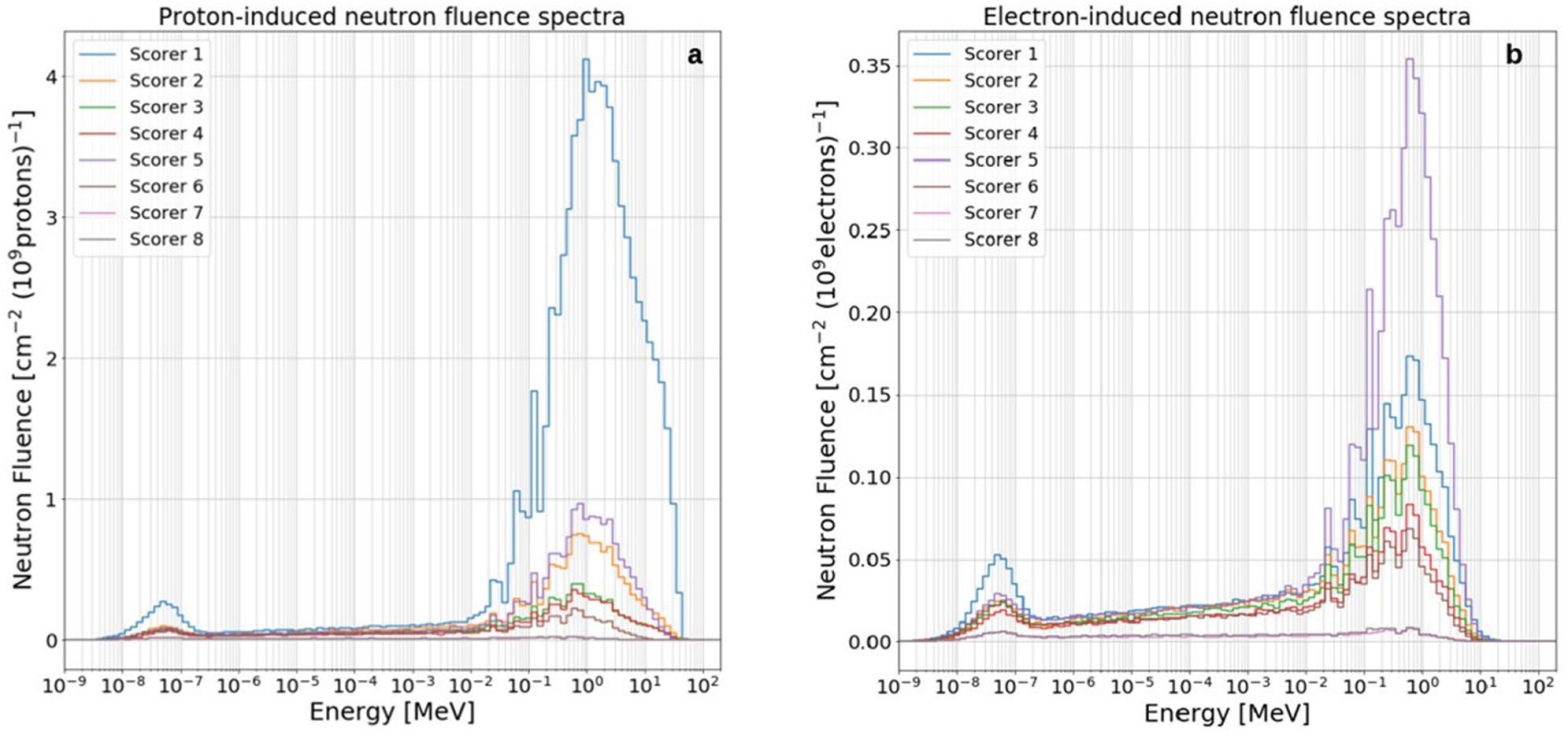
Proton- (**a**) and electron-induced (**b**) neutron fluence spectra for all scorers inside the LION cave (see Fig. 2) using Ma2019 input parameters. Values are normalized to 10^9^ primary particles.

In Fig. 8, the Ma2019 proton- and electron-induced secondary neutron spectra are plotted for all scorers inside the LION cave. Neutron spectra are characterized by a well-defined evaporation peak (centered at around 1 MeV) visible in all scored positions both for proton and electron source terms. In addition, on the low-energy edge of the evaporation peak of almost all scorers, a clearly noticeable pattern of spikes is visible, which is independent from whether neutrons are produced by proton- or electron-induced reactions. These spikes come from the neutron reaction cross section profiles, that for most of materials (e.g. ^27^Al of the vacuum chamber walls or ^16^O present the air) shows a resonance region typically ranging from a few keV to a few MeV. The interaction probability of a neutron passing through a medium is highly enhanced if it has an energy equal or close to one of the resonances of the medium crossed, resulting in an increased removal probability for neutrons with resonance energy. Clearly visible in most neutron spectra are the 35, 90 and 140 keV neutron elastic cross section resonances of ^27^Al and the elastic cross section resonance of ^16^O at about 430 keV.

Focusing on the proton-induced neutron spectra (Fig. 8a), in particular for scorer 1 (but also for scorers 2, 3 and 4) a slightly visible high-energy neutron component appears, more in the form of a tail rather than a clear peak. This is mainly the result of the interaction of highly energetic protons with the QPs and the air.

Apart from scorer 1, remarkably high is the neutron fluence at position 5, the closest to the QPs. No clear high-energy neutron component is present for this position because high-energy neutrons are mostly peak-forwarded, while scorer 5 lies at 90° with respect to the proton propagation direction.

Considering the electron-induced neutron spectra (Fig. 8b), one can clearly see that the highest neutron fluence is reached for scorer 5 rather than scorer 1. This might be due to the fact that more bremsstrahlung photons get produced in the QPs, which then initiate photo-nuclear reactions from that location, rather than in the beam dump, and since scorer 5 is closer than scorer 1 to the QPs position, this leads to a higher neutron fluence in 5 rather than in 1.

The contribution to the total neutron fluence for scorers 1 to 8 due to electrons, *f*_*e*_, has its minimum for position 1 (5%) and its maximum for position 5, 7 and 8 (26%). This can be calculated according to the following equation: $f_{e} = \Psi_{n,e} /(\Psi_{n,e} + \Psi_{n.p} )$, where $\Psi_{n,e}$ and $\Psi_{n,p}$ are electron- and proton-induced neutron fluences, respectively.

#### Wagner2016 p^+^ and e^−^ sources

**Figure 9:**
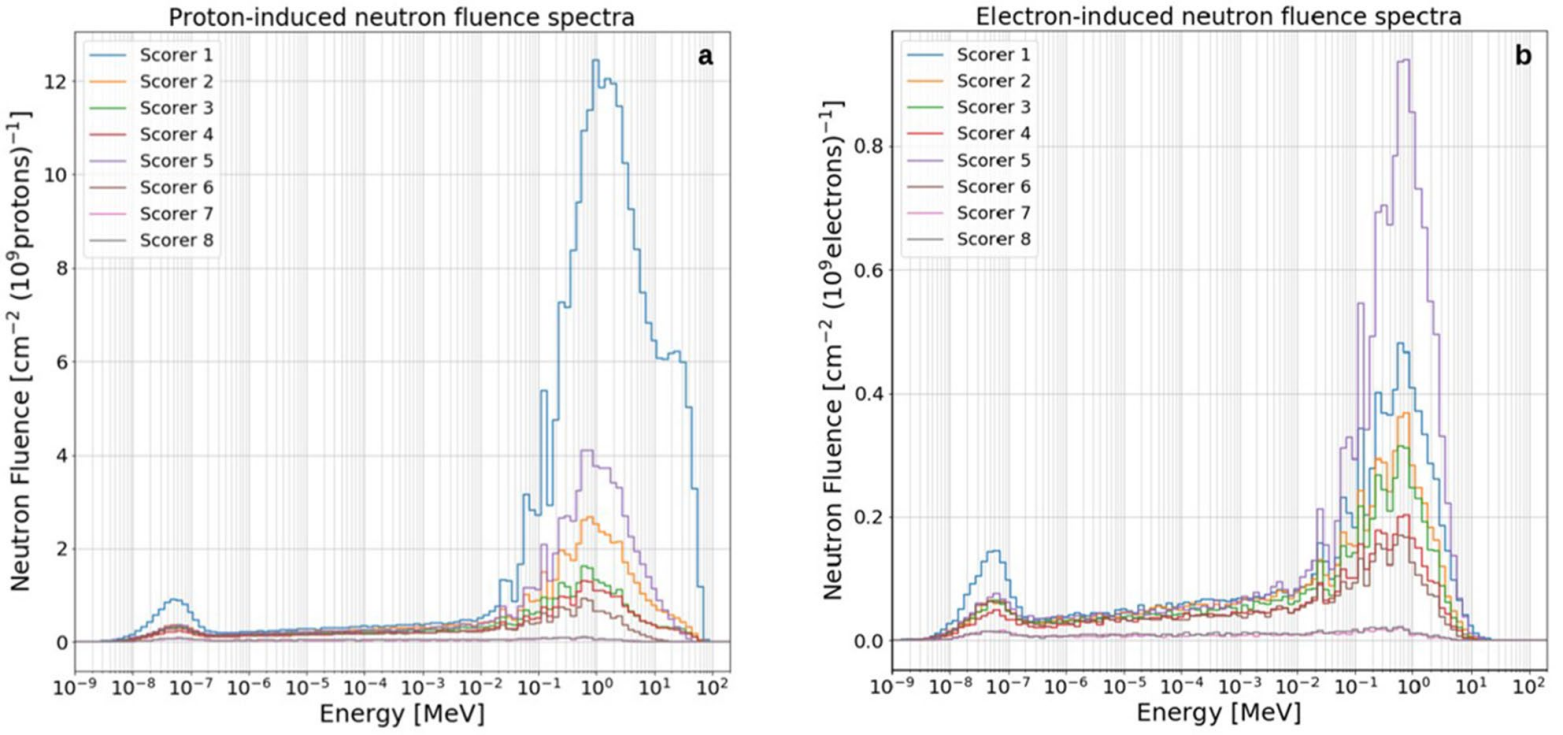
Proton- (**a**) and electron-induced (**b**) neutron fluence spectra for all scorers inside the LION cave (Fig. 2) using Wagner2016 input parameters. Values are normalized to 10^9^ primary particles.

As expected, secondary neutron spectra produced using Wagner2016 primary proton and electron source terms show higher neutron fluence per 10^9^ primary particles compared to those using Ma2019 and Zeil2010. With reference to Fig. 9, it is visible that both electron and proton-induced neutron spectra show well defined thermal and evaporation peaks. A more pronounced high-energy neutron component appears for scorers 1, 2, 3 and 4 due to the interaction of the high-energetic proton component with QPs and air. Additional MC simulations showed that in the absence of air this high energy peak is strongly reduced. The high-energy peak is reduced to a high-energy tail in other scored positions, for example 5 and 6, because of geometrical reasons.

As it happens with Ma2019 spectrum, the highest neutron fluence produced by electrons is registered for scorer 5. The contribution to the total neutron fluence due to electrons has its minimum for position 1 (4%) and its maximum for position 8 (18%).

### Dose analysis

#### Neutron dose

As expected, the neutron dose per 10^9^ primaries (both considering protons and electrons) in all scored positions inside the LION cave (scorers 1 to 8) increases with increasing the intensity of the source terms.

**Figure 10:**
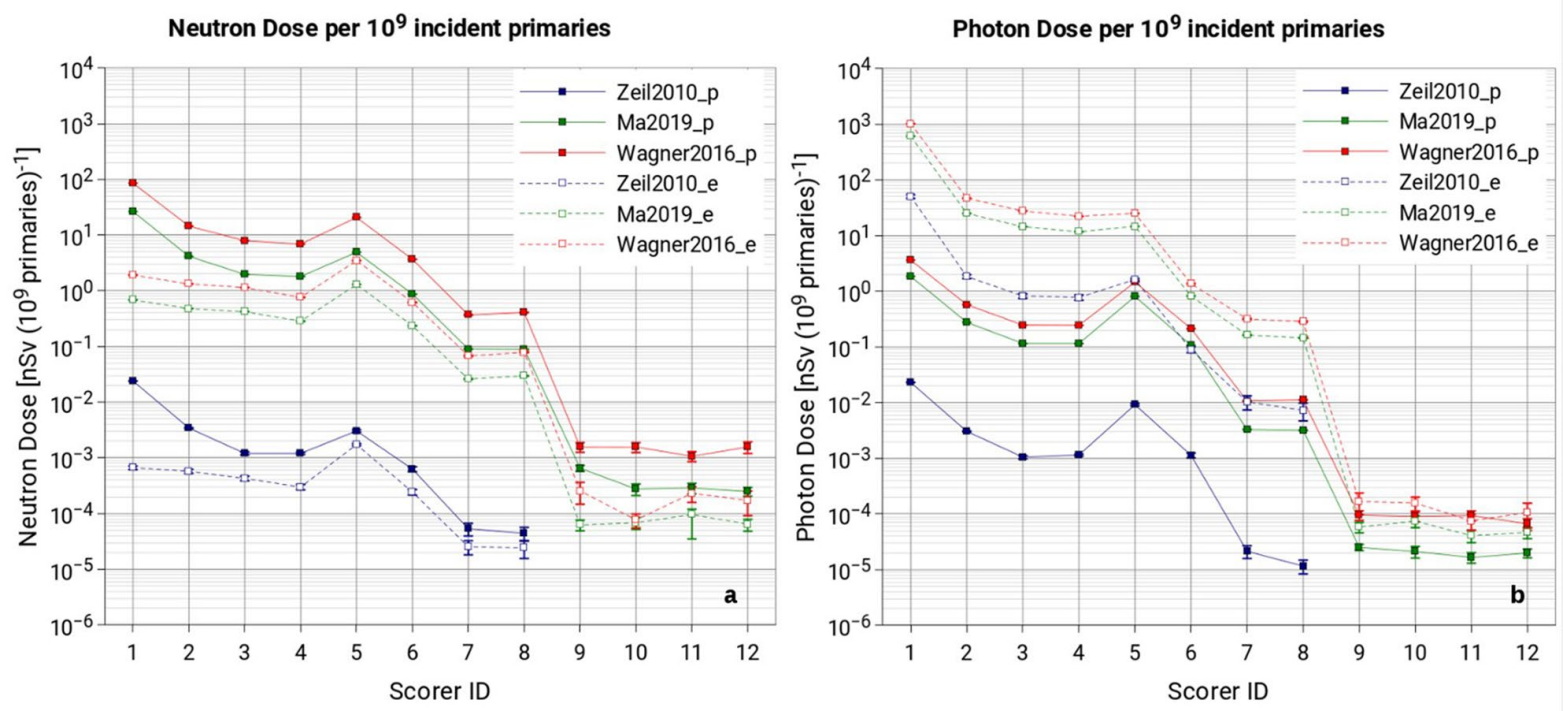
Neutron doses due to protons and electrons (**a**) and photon doses due to protons and electrons (**b**). Full markers represent the proton-induced component while open markers the electron-induced component. Data are expressed in terms of ambient dose equivalent and normalized to 10^9^ incident primary particles. The uncertainty is depicted by vertical bars, where missing, it is because uncertainties are smaller than the marker itself. To better visualize the data, data points have been connected with straight lines.

As visible in Fig. 10a and summarized in Tables S1, S2 and S3 of the Supplementary Material, higher dose values can be found close to those positions where most of the protons interact (i.e., scorers 1 to 5). The maximum simulated neutron dose is 85 nSv(10^9^protons)^−1^, registered in scorer 1 for Wagner2016 proton source. Scorer 5 shows a noticeably high dose per 10^9^ primaries for all employed primary source terms. This is direct consequence of the interaction of the D-fraction of laser-driven particles with the two QPs. This is particularly true for the electron-induced neutron dose, that has its maximum in scorer 5 (instead of scorer 1) for all employed electron sources.

Simulated neutron doses outside the LION cave are a few orders of magnitude lower than those calculated inside. Considering the proton-induced component to the neutron dose, values remain below 2 × 10^–3^ and 7 × 10^–4^ nSv(10^9^protons)^−1^ for Wagner2016 and Ma2019, respectively. While considering the electron-induced component, neutron dose is below 3 × 10^–4^ and 10^–4^ nSv(10^9^electrons)^−1^ for Wagner2016 and Ma2019, respectively. No neutrons have been detected outside using Zeil2010 primary proton and electron spectra. This is due to the already low neutron fluence inside the LION cave and the limited available computation time.

It has to be pointed out that the statistical uncertainties associated with total neutron doses outside the LION cave (ranging from 12 to 22%, see Supplementary Material) does not allow us to consider these values more than as merely indicative.

#### Photon dose

As shown in Fig. 10b, the maximum value of photon dose per 10^9^ primaries is 1.0 × 10^3^ nSv(10^9^electrons)^−1^, found in scorer 1 for Wagner2016 electron source. Compared to the neutron case, the photon dose distribution appears to be more forward-peaked. This can be explained by the fact that photon dose is dominated by bremsstrahlung photons and the bremsstrahlung photon emission is clearly forward-peaked already at about 1 MeV initial electron energy^37^. The proton-induced photon dose is a few orders of magnitude lower than the respective electron-induced component for all scorers and appears to be less forward-peaked. As said, photon radiation, when electrons are employed as projectiles, is mainly coming from *bremsstrahlung.* In contrast, when protons are employed, photon production is mainly due to prompt gamma emission as a consequence of nuclear relaxation following proton inelastic scattering or proton-induced nuclear reactions.

As it is for neutrons, simulated photon doses outside the LION cave are a few orders of magnitude lower than those calculated inside. Considering the proton-induced component to the photon dose, values remain below 10^–4^ and 3 × 10^–5^ nSv(10^9^protons)^−1^ for Wagner2016 and Ma2019, respectively. In contrast, considering the electron-induced component, photon dose is below 2 × 10^–4^ and 8 × 10^–5^ nSv(10^9^electrons)^−1^ for Wagner2016 and Ma2019, respectively. Similarly, to the neutron case, no photons have been detected using Zeil2010 primary proton and electron spectra.

Uncertainties associated to total photon doses outside the LION cave range from 5 to 20%. In analogy to what is mentioned discussing neutron doses outside the LION cave, these values are to be taken as indicative evaluations only.

## Discussion

By using three different scenarios characterized by different primary proton and electron spectra, it has been possible to analyze the production of secondary photons and neutrons for three different stages of the LION facility’s commissioning.

As expected, among all three source terms investigated within this study, the highest secondary particle fluences per 10^9^ primaries were found for those scorers that are close to where most of the primaries interact (scorers 1 to 5). In addition to this, the neutron and photon fluence per 10^9^ primaries increases for each position with increasing primary input parameters (e.g., higher cutoff energy or higher fraction of high energy particles).

**Figure 11:**
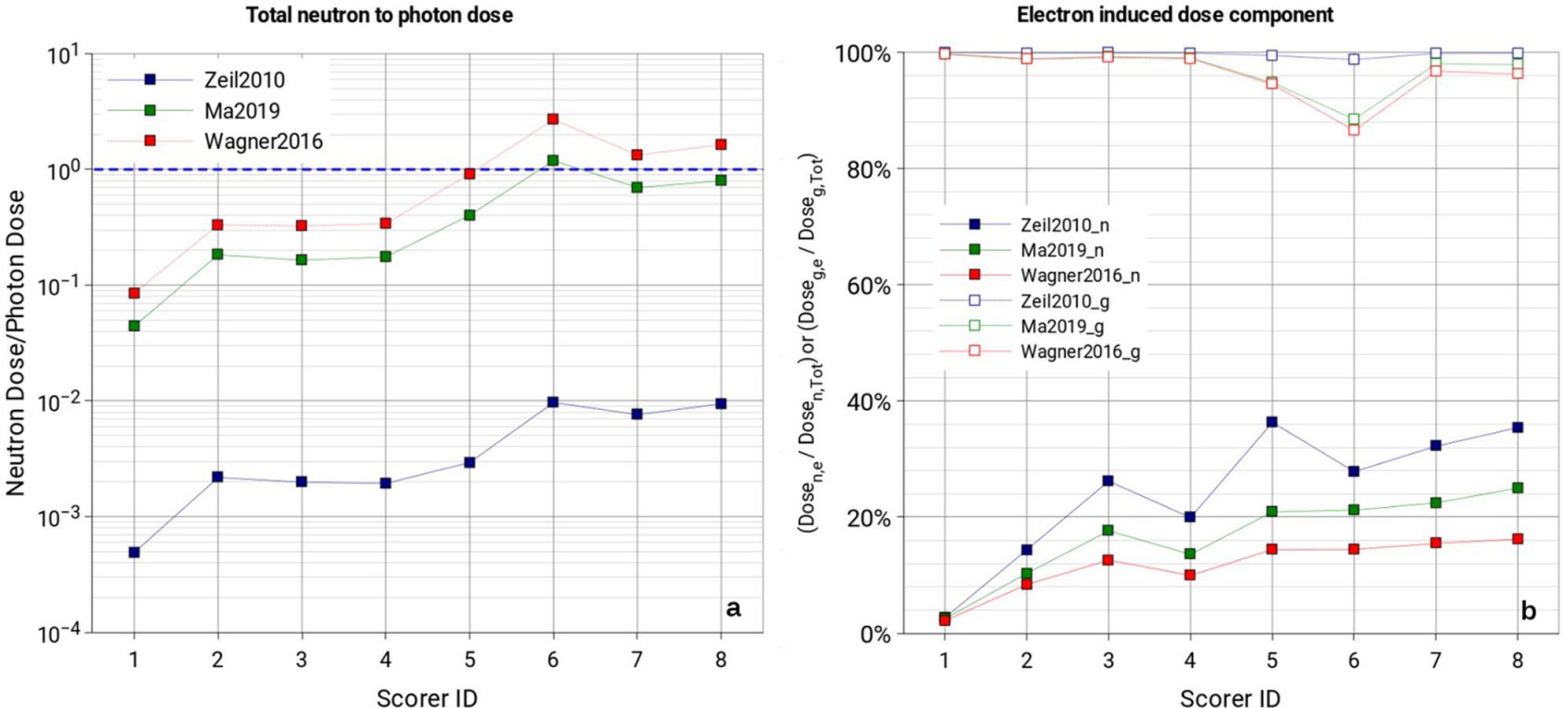
(**a**) Total neutron to total photon dose ratio (the blue dashed line lies at ratio = 1). (**b**) Electron-induced neutron dose fraction of the total neutron dose (full markers) and electron-induced photon dose fraction of the total photon dose (open markers). Values are displayed for all scored positions inside the LION cave (scorers 1–8). To guide the eye, data points have been linked using straight lines.

Looking at the two components of the secondary radiation field, we can say that, inside the experimental cave, the field is either dominated by photons or neutrons, depending on the source term used and the position in the room. As shown in Fig. 11a, for Zeil2010 the ratio of total neutron dose over total photon dose remains below 10^–2^ for all scored positions. These changes using Ma2019 and Wagner2016 source terms, for which scorers 6, 7 and 8 show neutron over photon dose ratios close or above 1. This can be explained by the fact that Ma2019 and Wagner2016 have a significant fraction of protons and electrons above the respective neutron production thresholds, while for Zeil2010 only a minor fraction of primaries has enough energy to induce neutron production. From Fig. 11a it is also evident that regions far from the hot spots and positions behind the beam dump (scorers 6, 7 and 8) show a higher neutron over photon dose ratio for all employed primary source terms. This comes from the fact that neutrons, with respect to photons, travel more easily throughout the room due to scattering events with the room walls and room elements.

Figure 11b shows the electron-induced fraction of the total neutron (full markers) and photon dose (open markers), for all scorers inside the LION cave. It is clearly visible that the electron-induced neutron dose fraction remains for all positions below 40% and decreases with increasing the primary source intensity. Highest values of the electron-induced neutron fraction are found for Zeil2010 and lowest values for Wagner2016. Such behavior could be explained by the different energy-dependent (p,n) and (γ,n) production cross sections on ^27^Al, which makes up the major elemental component of the QPs protection layer (where most of secondary particles are produced), combined with the fact that the Zeil2010 proton spectrum has a cutoff energy below the main peak of the (p,n) reaction on ^27^Al, therefore, it is reasonable to expect a large increase in proton-induced neutron production when using spectra with higher cutoff energies compared to Zeil2010 (i.e., Ma2019 or Wagner2016).

This plot indicates not only that the production of neutrons is mainly due to protons, but that the higher the source intensity and energy the higher the contribution of protons to the production of secondary neutrons, suggesting that, for even higher source intensities and energies, the contribution of electrons to the neutron production could even become negligible.

In contrast, the photon production is almost entirely due to the electron component of the source term. The contribution of protons to the production of secondary photons slightly increases with increasing source intensity and energy specially for scorer 6 (open markers, Fig. 11b).

In this work it has been assumed that only 10% of particles is able to pass through the two QPs and therefore, the remaining 90% inevitably interacts with the first QP. Experimentally speaking this might not be always the case and, consequently, it is worth looking to what happens when changing the proportion between F- and D-fraction of the primary source term.

**Figure 12:**
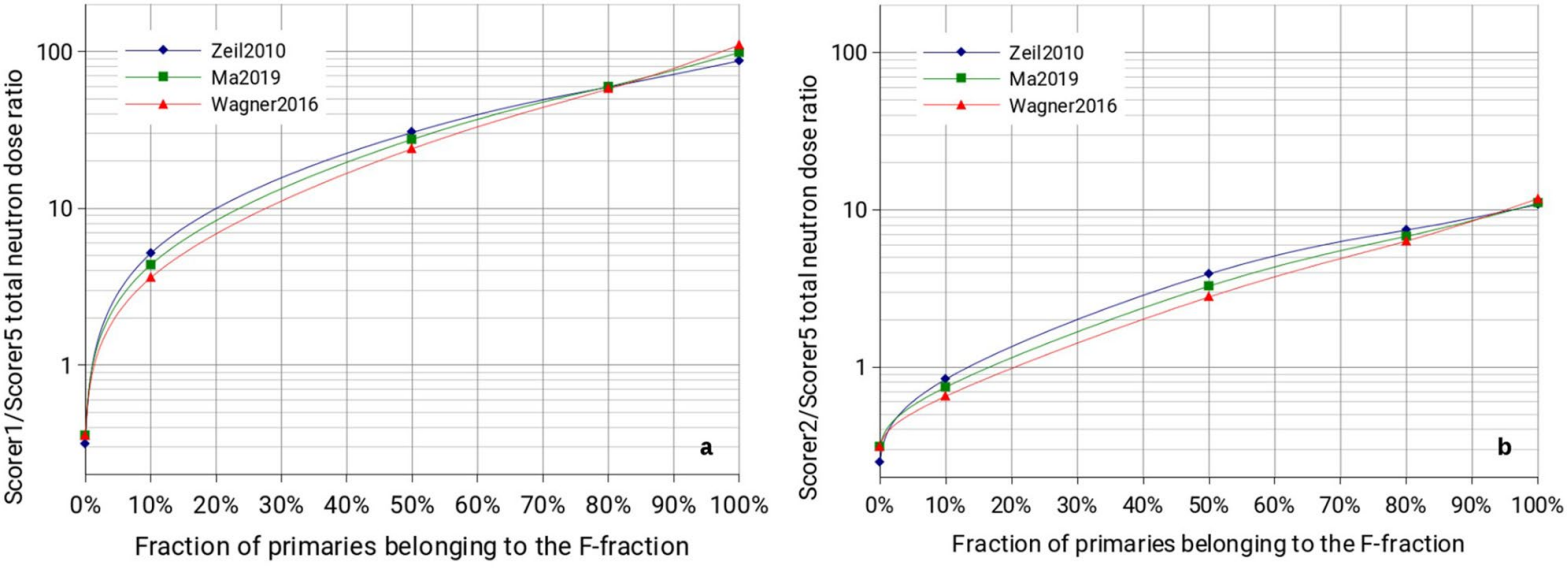
(**a**) Ratio between the neutron dose calculated in scorer 1 and scorer 5 depending on the fraction of primaries belonging to the F-fraction. (**b**) Ratio between the neutron dose calculated in scorer 2 and scorer 5 depending on the fraction of primaries belonging to the F-fraction. To guide the eye, a spline interpolation has been applied.

Figure 12a shows how the ratio of the total neutron dose between Scorer 1 and Scorer 5, $D_{1}^{n} /D_{5}^{n}$ (where *n* stands for *neutron* and the number refers to the scorer ID), varies by increasing the fraction of primaries belonging to the F-fraction (expressed in % of the total). For clarity, 0% means that no primary got focused, while 100% means that all primaries got focused. Figure 12b shows the same information considering for the ratio scorer 2 instead of scorer 1. It is clearly visible that there is a strong dependence of $D_{1}^{n} /D_{5}^{n}$ and $D_{2}^{n} /D_{5}^{n}$ on the fraction of primaries belonging to the F-fraction, from a minimum of about 0.1 to a maximum of about 100. This is no surprise, given that scorer 1 and 2 are clearly more affected by the F-fraction of primaries then by the D-fraction because of their position, while scorer 5 is less affected by the F-fraction. It needs also to be noted that these ratios appear to be almost independent from the primary source term used (and also on the number of primaries produced, being a ratio), suggesting that knowing experimentally the ratio of the secondary neutron dose for scorers 1, 2 and 5 might already give a hint on the real proportion between F-fraction and D-fraction experimentally produced. This consideration shows an attractive application of neutron (dose) measurements at LION (and more generally at any laser-driven ion sources) as a tool to acquire knowledge on the primary source angular distribution. As an example, if it were experimentally determined that the neutron dose measured in scorer 2 is twice as high as the one measured by scorer 5, we could infer that the number of protons belonging to the F-fraction ranges from 30 to 40% even not knowing exactly what primary proton spectrum or cutoff energy were used (note that the variation in terms of cutoff energy and steepness of the proton spectrum between Zeil2010 and Wagner2016 are much greater than the expected fluctuation of these parameters from shot to shot).

With regards to radiation protection, outside the LION cave, maximum total neutron and photon doses were found to be below 1.8 × 10^–3^ nSv (10^9^ primaries)^−1^ and 2.6 × 10^–4^ nSv (10^9^ primaries)^−1^ respectively. Assuming a repetition frequency of 1 Hz (and bunches composed by 10^9^ primaries, as in Englbrecht et al.^9^) maximum dose rates of 6.5 and 0.9 nSv/h can be calculated for neutrons and photons, respectively. Although, as mentioned earlier, these values have to be taken only as indicative evaluations given the large associated uncertainties, these results are well below 0.5 μSv/h (dose limit specified for unclassified areas, as is the corridor where scorers 9 and 10 are located^9^) and do not raise any radiation protection concerns. Proton-induced neutron dose rates found by Englbrecht et al. for similar locations outside the LION cave are on the average 16 times larger those derived here by using the Wagner2016 proton source term (again assuming same repetition frequency and number of primary protons per bunch). A larger difference could have been expected considering that Wagner2016 proton source is exponentially shaped with cutoff energy of 86 MeV, while Englbrecht et al.’s source has a flat spectrum from 10 to 200 MeV, and the fact that the scorers used in the present study are placed at the height of the double floor (about 65 cm above laser openings) while Englbrecht et al.’s scorers are at the very exit of laser openings. It has to be noted, however, that most of neutrons in the present study originate from the QPs, rather then from the beam dump (as instead was the case in Englbrecht et al. work). The QPs are closer to the laser openings compared to the beam dump and, therefore, neutrons emitted at that location can more easily leak through them, compared to those neutrons coming from the beam dump.

Lastly, despite the fact that this work and the one presented by Fan et al.^7^ describe different facilities, it is possible to compare both studies in terms of neutron dose per primary between a few scorers presented in this work and some of the ones described by Fan et al.’s work, thanks to their similar positions with respect to the source term location. The doses per primary proton at 1 m distance from the vacuum chamber hosting the laser-target interaction site showed by Fan et al., in the case of no additional shielding (only vacuum chamber structure), range from about 2.9 × 10^–8^ to 1.2 × 10^–7^ nSv/p^+^. If we consider only the D-fraction (in order to be as close as possible to what is described by Fan et al., where neutrons were produced only by the interactions with components inside the vacuum chamber*)* of the Wagner2016 primary proton source, we have neutron doses per primary ranging from 3.9 × 10^–9^ to 2.2 × 10^–8^ nSv/p^+^ (considering only scorers in the proximity of the vacuum chamber, i.e., scorers 1 to 6). The roughly one order of magnitude difference between the two results can be explained by the different cutoff energy: for Wagner2016 it lies at about 90 MeV while for Fan et al. goes up to about 300 MeV. In order to get a closer comparison, a Fan-like proton spectrum would be necessary as input parameter.

## Conclusions and outlook

Geant4 Monte Carlo simulations have been performed in order to assess the expected secondary neutron fluence and dose at different locations around the LION experiment during its commissioning. To achieve this goal, three different scenarios have been simulated using proton spectra taken from literature as input parameters. To fully characterize the LION source, also electrons have been included using a simple exponential model of the electron energy distribution. Moreover, a simplified geometrical model of the proton and electron source (composed of divergent and focused fractions) has been proposed in order to reduce the simulation complexity, yet deriving realistic results.

As expected, maximum secondary neutron and photon doses per 10^9^ primaries have been found at positions 1 and 5 close to those elements where most of the laser-accelerated particles interact (namely, beam dump and QPs). This fact underlines that a detailed description of the elements present inside the vacuum chamber (and not only the ones present in the LION cave) is of primary importance for characterizing the secondary radiation field present at this facility. Maximum neutron and photon doses reached 85 nSv (10^9^ primaries)^−1^ and 1.0 × 10^3^ nSv (10^9^ primaries)^−1^ respectively, for scorer 1 and Wagner2016 primary source term.

Thanks to this analysis it has been also found that the radiation environment is initially dominated by photons, when low intensity sources are employed (i.e., Zeil2010), but when sources with higher intensity are employed (i.e., Ma2019 and Wagner2016), neutrons dominate the radiation field.

Interestingly we found that an experimental evaluation of the ratio of the neutron dose measured in forward direction (scorer 1 or 2) and on the side (scorer 5) might give a good estimate of the proportion of particles that pass through obstacles (in our case QPs) and those that are stopped in these obstacles, almost independently from the primary source term spectrum and number of primaries produced. This could serve as a diagnostic tool to better characterize the primary proton source.

Natural next step of this work is an evaluation of the performances of available neutron and photon detection techniques in this radiation environment, followed by an experimental verification of the simulated results by performing neutron and photon radiation mappings.

In addition, to generalize the findings of this work to other facilities, the impact of using different materials for the main components (such as vacuum chamber, QPs and beam dump) on the production of the secondary radiation field is planned in the near future.

Moreover, the energy-independent two-components source term model used in this work could be further improved in order to derive a more realistic source model. This could be done in a group-wise way, further dividing the source term into a few energetic groups to which different probabilities for F-fraction and D-fraction are applied or by global simulations that involve electric and magnetic fields of beam guide components.

For a more complete radiological characterization of the LION experiment, computational studies of the residual induced activation, not covered in this manuscript, will also be performed. However, computational results by Florescu et al.^38^ show that the residual dose rate due to activation at a few cm from the surface of an aluminum-made vacuum chamber is comparable with the average natural outdoor radiation background already after three minutes of cooling time, when using 100 MeV mono-energetic protons and 10^14^ protons per bunch. This result suggests that the contribution of activation to the total dose should be minimal, considering the primary source terms employed in this study.

## Supplementary Information

Supplementary Tables.
