# Supplementary material for: Geant4 Monte Carlo simulation study of the secondary radiation fields at the laser-driven ion source LION
Source: Sci Rep. 2021 Dec 24;11:24418. doi: 10.1038/s41598-021-03897-2 (PMC8709851; doi:10.1038/s41598-021-03897-2)
Supplement: Supplementary file 1 — Supplementary Tables. [file 41598_2021_3897_MOESM1_ESM.pdf]

Geant4 Monte Carlo simulation study of the secondary radiation fields at the laser-driven ion source LION: **supplementary material**

M. Tisi[1,*], V. Mares[1], J. Schreiber[2,3], F. S. Englbrecht[2] and W. Rühm[1]

1. Helmholtz Zentrum München, Institute of Radiation Medicine, Neuherberg, Germany
2. Ludwig-Maximilians-Universität, Faculty of Physics, Department of Medical Physics, Garching bei München, Germany
3. Max-Plank-Institute for Quantum Optics, Garching bei München, Germany

*corresponding author, email: marco.tisi@helmholtz-muenchen.de

A. Tables of neutron and photon dose normalized to 10^9 incident primary particles

Neutron and photon doses per 10^9 primaries are listed in table S1, S2 and S3 for Zeil2010, Ma2019 and Wagner2016primary proton and electron sources respectively. Since 120 cores have been used to run these simulations, statistical uncertainties have been calculated using the standard error of the mean. They are reported as relative (%) values in a separated column.

Zeil2010

**Zeil2010**	**Neutron dose [nSv (10^9 primaries)$^{-1}$]**						**Photon dose [nSv (10^9 primaries)$^{-1}$]**					
**ID**	**p^+**	**σ_{rel}**	**e^-**	**σ_{rel}**	**Sum**	**σ_{rel}**	**p^+**	**σ_{rel}**	**e^-**	**σ_{rel}**	**Sum**	**σ_{rel}**
1	2.4×10^{-2}	0.7%	6.7×10^{-4}	7.6%	2.5×10^{-2}	0.7%	2.3×10^{-2}	0.3%	5.1×10^{1}	0.1%	5.1×10^{1}	0.1%
2	3.4×10^{-3}	2.1%	5.8×10^{-4}	8.2%	4.0×10^{-3}	2.1%	3.0×10^{-3}	0.8%	1.8×10^{0}	0.5%	1.8×10^{0}	0.5%
3	1.2×10^{-3}	6.5%	4.3×10^{-4}	9.5%	1.6×10^{-3}	5.4%	1.1×10^{-3}	2.4%	8.2×10^{-1}	0.7%	8.3×10^{-1}	0.7%
4	1.2×10^{-3}	4.6%	3.0×10^{-4}	11.0%	1.5×10^{-3}	4.3%	1.1×10^{-3}	1.7%	7.8×10^{-1}	0.8%	7.8×10^{-1}	0.8%
5	3.0×10^{-3}	4.9%	1.7×10^{-3}	4.3%	4.8×10^{-3}	3.5%	9.2×10^{-3}	0.9%	1.6×10^{0}	0.4%	1.6×10^{0}	0.4%
6	6.3×10^{-4}	10.4%	2.4×10^{-4}	11.6%	8.7×10^{-4}	8.2%	1.1×10^{-3}	2.5%	8.9×10^{-2}	1.2%	9.0×10^{-2}	1.2%
7	5.3×10^{-5}	25.7%	2.5×10^{-5}	28.5%	7.9×10^{-5}	19.7%	2.1×10^{-5}	8.9%	1.0×10^{-2}	4.2%	1.0×10^{-2}	4.2%
8	4.4×10^{-5}	27.8%	2.4×10^{-5}	35.3%	6.9×10^{-5}	21.9%	1.2×10^{-5}	6.8%	7.3×10^{-3}	4.7%	7.3×10^{-3}	4.7%
9	0	-	0	-	0	-	0	-	0	-	0	-
10	0	-	0	-	0	-	0	-	0	-	0	-
11	0	-	0	-	0	-	0	-	0	-	0	-
12	0	-	0	-	0	-	0	-	0	-	0	-

Table S1: Neutron and photon doses given in [nSv (10^9 primaries)$^{-1}$] for Zeil2010 proton and electron sources.

Ma2019

Ma2019	Neutron dose [nSv (10^9 primaries)$^{-1}$]						Photon dose [nSv (10^9 primaries)$^{-1}$]					
ID	p^+	σ_{rel}	e^-	σ_{rel}	Sum	σ_{rel}	p^+	σ_{rel}	e^-	σ_{rel}	Sum	σ_{rel}
1	2.6×10^{1}	0.1%	6.8×10^{-1}	1.1%	2.7×10^{1}	0.1%	1.8×10^{0}	0.1%	6.1×10^{2}	0.0%	6.1×10^{2}	0.0%
2	4.2×10^{0}	0.2%	4.8×10^{-1}	1.6%	4.6×10^{0}	0.2%	2.8×10^{-1}	0.1%	2.5×10^{1}	0.1%	2.5×10^{1}	0.1%
3	2.0×10^{0}	0.3%	4.1×10^{-1}	1.9%	2.4×10^{0}	0.3%	1.2×10^{-1}	0.2%	1.4×10^{1}	0.1%	1.5×10^{1}	0.1%
4	1.8×10^{0}	0.3%	2.8×10^{-1}	2.2%	2.1×10^{0}	0.3%	1.2×10^{-1}	0.1%	1.2×10^{1}	0.1%	1.2×10^{1}	0.1%
5	4.9×10^{0}	0.2%	1.3×10^{0}	1.0%	6.2×10^{0}	0.2%	8.1×10^{-1}	0.1%	1.5×10^{1}	0.1%	1.5×10^{1}	0.1%
6	8.7×10^{-1}	0.4%	2.2×10^{-1}	2.3%	1.1×10^{0}	0.4%	1.1×10^{-1}	0.2%	8.2×10^{-1}	0.2%	9.3×10^{-1}	0.2%
7	9.0×10^{-2}	1.1%	2.6×10^{-2}	6.8%	1.2×10^{-1}	0.9%	3.3×10^{-3}	0.9%	1.6×10^{-1}	0.5%	1.7×10^{-1}	0.5%
8	8.9×10^{-2}	1.4%	3.0×10^{-2}	5.5%	1.2×10^{-1}	1.1%	3.2×10^{-3}	0.8%	1.5×10^{-1}	0.6%	1.5×10^{-1}	0.6%
9	6.5×10^{-4}	12.7%	6.3×10^{-5}	21.6%	7.2×10^{-4}	11.7%	2.5×10^{-5}	9.4%	5.8×10^{-5}	3.6%	8.4×10^{-5}	3.8%
10	2.8×10^{-4}	23.4%	6.9×10^{-5}	24.0%	3.4×10^{-4}	19.3%	2.1×10^{-5}	10.7%	7.5×10^{-5}	2.8%	9.6×10^{-5}	3.2%
11	2.9×10^{-4}	21.4%	9.6×10^{-5}	25.0%	3.8×10^{-4}	17.2%	1.7×10^{-5}	11.1%	4.1×10^{-5}	4.7%	5.7×10^{-5}	4.6%
12	2.5×10^{-4}	18.1%	6.5×10^{-5}	22.3%	3.1×10^{-4}	15.1%	2.0×10^{-5}	11.6%	4.6×10^{-5}	3.7%	6.6×10^{-5}	4.3%

Table S2: Neutron and photon doses given in [nSv (10^9 primaries)$^{-1}$] for Ma2019 proton and electron sources.

Wagner2016

Wagner2016	Neutron dose [nSv (10^9 primaries)$^{-1}$]						Photon dose [nSv (10^9 primaries)$^{-1}$]					
ID	p^+	σ_{rel}	e^-	σ_{rel}	Sum	σ_{rel}	p^+	σ_{rel}	e^-	σ_{rel}	Sum	σ_{rel}
1	8.5×10^{1}	0.1%	1.9×10^{0}	0.5%	8.7×10^{1}	0.1%	3.6×10^{0}	0.0%	1.0×10^{3}	0.0%	1.0×10^{3}	0.0%
2	1.4×10^{1}	0.2%	1.3×10^{0}	0.7%	1.6×10^{1}	0.2%	5.8×10^{-1}	0.1%	4.7×10^{1}	0.1%	4.8×10^{1}	0.1%
3	7.9×10^{0}	0.3%	1.1×10^{0}	0.7%	9.1×10^{0}	0.3%	2.5×10^{-1}	0.3%	2.8×10^{1}	0.1%	2.8×10^{1}	0.1%
4	6.9×10^{0}	0.3%	7.6×10^{-1}	0.8%	7.6×10^{0}	0.3%	2.4×10^{-1}	0.2%	2.2×10^{1}	0.1%	2.2×10^{1}	0.1%
5	2.1×10^{1}	0.2%	3.5×10^{0}	0.5%	2.4×10^{1}	0.2%	1.5×10^{0}	0.1%	2.5×10^{1}	0.1%	2.6×10^{1}	0.1%
6	3.7×10^{0}	0.5%	6.2×10^{-1}	1.1%	4.3×10^{0}	0.5%	2.1×10^{-1}	0.3%	1.4×10^{0}	0.2%	1.6×10^{0}	0.2%
7	3.7×10^{-1}	1.6%	6.8×10^{-2}	2.6%	4.4×10^{-1}	1.4%	1.1×10^{-2}	1.3%	3.2×10^{-1}	0.5%	3.3×10^{-1}	0.4%
8	4.1×10^{-1}	1.3%	7.9×10^{-2}	2.3%	4.9×10^{-1}	1.2%	1.1×10^{-2}	1.3%	2.9×10^{-1}	0.6%	3.0×10^{-1}	0.5%
9	1.6×10^{-3}	19.6%	2.6×10^{-4}	42.2%	1.8×10^{-3}	17.9%	9.5×10^{-5}	11.6%	1.7×10^{-4}	22.4%	2.6×10^{-4}	14.9%
10	1.6×10^{-3}	20.1%	7.7×10^{-5}	27.9%	1.6×10^{-3}	19.2%	9.1×10^{-5}	9.0%	1.6×10^{-4}	20.5%	2.5×10^{-4}	13.4%
11	1.1×10^{-3}	20.3%	2.3×10^{-4}	31.7%	1.3×10^{-3}	17.6%	9.4×10^{-5}	11.3%	7.6×10^{-5}	15.6%	1.7×10^{-4}	9.4%
12	1.6×10^{-3}	23.4%	1.7×10^{-4}	46.8%	1.7×10^{-3}	21.6%	6.6×10^{-5}	13.7%	1.1×10^{-4}	21.6%	1.7×10^{-4}	14.3%

Table S3: Neutron and photon doses given in [nSv (10^9 primaries)$^{-1}$] for Wagner2016 proton and electron sources.
